# Triglycerides to high-density lipoprotein cholesterol ratio and its association with periodontitis—a systematic review

**DOI:** 10.1038/s41405-025-00381-1

**Published:** 2025-12-04

**Authors:** Shashikiran Shanmugasundaram, Shaswata Karmakar, Ramya Arangaraju, Ragavi Alagarsamy

**Affiliations:** 1 Department of Periodontology, Manipal College of Dental Sciences, Manipal, Manipal Academy of Higher Education, Manipal, Karnataka India; 2 Department of Oral and Maxillofacial Surgery, Yenepoya Dental College, Yenepoya (deemed to be university), Mangalore, Karnataka India; 3 Department of Oral and Maxillofacial Surgery, Maulana Azad Institute of Dental Sciences, New Delhi, India

**Keywords:** Periodontitis, Periodontitis, Dental epidemiology

## Abstract

**Background and aim:**

Metabolic health is closely related to periodontal health. Obesity, metabolic syndrome and diabetes mellitus are bidirectionally associated with periodontitis. The triglycerides-to-high-density lipoprotein cholesterol ratio (Ty/HDLc ratio) has emerged as a novel biomarker that can predict the risk of several chronic metabolic diseases. We conducted this systematic review to consolidate the available evidence regarding the association between the Ty/HDLc ratio and periodontitis.

**Methods:**

We systematically searched PubMed, SCOPUS, Embase, and Web of Science databases for studies that assessed the Ty/HDLc ratio and its association with periodontitis till May 2025. No date or language restrictions were applied. We included all observational studies in adults and adolescents and excluded interventional studies, case reports, and animal studies. Study quality was assessed using the Joanna Briggs Institute critical appraisal checklist.

**Results:**

Systematic screening of the search results yielded 7 studies that met our eligibility criteria. Sample sizes varied from 69 to 13,584 individuals aged 17 to 53 years across diverse population groups from Brazil, Mexico, South Korea, and Taiwan. All included studies were rated as having a low risk of bias. Despite demographic differences, all studies found a consistent positive association between the Ty/HDLc ratio and periodontitis prevalence and/or severity.

**Conclusion:**

The findings of this review suggest that the serum Ty/HDLc ratio is positively associated with periodontitis prevalence and could be a promising biomarker that is simple, practical, cost-effective, routinely measured, and easily calculable, and holds the potential to be an indirect risk marker for periodontitis. Future research should focus on establishing the temporal relationship and establishing a definitive, gender-specific cut-off for periodontal risk assessment.

## Introduction

Periodontitis is one of the most prevalent chronic non-communicable diseases, with a global prevalence of 19% and manifests in the oral cavity. It has led to a severe public health crisis that has a significant impact on the quality of life and well-being of an individual [1, 2]. Chronic inflammation around teeth can cause progressive loss of soft and hard tissues, eventually leading to tooth loss [3, 4]. Periodontitis also has a widespread systemic impact on various organ systems through chronic low-grade systemic inflammation and increases the risk of several systemic diseases [5–8]. Various systemic diseases and conditions can increase the risk of periodontitis; periodontitis has a three-way relationship to metabolic diseases like obesity and type 2 diabetes mellitus (T2DM) through low-grade systemic inflammation, with shared risk factors and often resulting in multimorbidity [9, 10].

Given the strong relationship between metabolic diseases and periodontitis, it is logical to infer that the overall metabolic health of an individual plays an important role in the initiation and progression of periodontitis [9]. Metabolic health is reflected by how efficiently the body processes caloric energy for various physiologic functions; chronic dysregulation of metabolic health due to altered carbohydrate and lipid metabolism manifests as dyslipidemia, obesity, insulin resistance, and hyperglycemia, and results in a systemic pro-inflammatory state [11, 12]. Potential consequences of long-term metabolic dysregulation are metabolic syndrome, T2DM, CVD and periodontitis [9]. Theoretically, any biomarker that accurately reflects the underlying metabolic health status might hold the potential to predict the risk of co-morbidities associated with metabolic dysregulation, including periodontitis. And, given the high prevalence of periodontitis, there is an urgent need for effective risk markers that can identify individuals at risk to optimise primary prevention.

Lipid profile is a valuable tool to assess metabolic and cardiovascular health. Lipid metabolism is an intricately balanced process that is vital for optimal health. An abnormal lipid profile due to dysregulated lipid metabolism (dyslipidemia) is associated with metabolic syndrome, CVD, and T2DM [13–15]. A comprehensive meta-analysis found that periodontitis and dyslipidemia were associated bi-directionally [16]. Among the lipid profile parameters, serum triglycerides (Ty) and high-density lipoprotein-cholesterol (HDLc) have attracted attention for their crucial roles in dyslipidemia and metabolic syndrome [17]. A deranged lipid profile—high triglycerides (>150 mg/dl) and low HDL cholesterol (<40 mg/dl)—is one of the criteria to define metabolic syndrome [18]. Recently, this criterion has been adapted and studied as the serum triglycerides-to-serum high-density lipoprotein cholesterol ratio (Ty/HDLc ratio), deemed to be an effective risk marker for various chronic diseases [19].

The Ty/HDLc ratio has emerged as an indirect biomarker that predicts insulin resistance [20–24]. The ratio also predicts metabolic syndrome [17, 19, 25]. It is important to note that insulin resistance, dyslipidemia, and central obesity are important aspects of metabolic syndrome, all of which are closely associated with periodontal inflammation [5, 9, 10]. Extensive evidence also shows that the Ty/HDLc ratio is a risk marker for CVD and cerebrovascular disease [19, 24, 26–31]. It is important to note that periodontitis is associated with CVD and cerebrovascular disease through low-grade systemic inflammation [5, 32, 33]. Overall, the Ty/HDLc ratio can potentially serve as a reliable risk indicator for several interrelated chronic non-communicable diseases and conditions, also indicating underlying deficiencies in an individual’s metabolic health. Periodontitis is an important link in the interconnected web of NCDs and is shown to be associated with dyslipidemia. Hence, the Ty/HDLc ratio might be a promising biomarker that can serve as a simple and effective risk indicator for periodontitis that can aid in better primary prevention. Despite promising associations, biomarkers for metabolic syndrome, such as the Ty/HDLc ratio, face challenges, including population-specific and gender-specific variability, a lack of standardised thresholds, and residual confounding from medication use and unmeasured lifestyle factors [34, 35]. These limitations underscore the need for further validation in the context of oral health.

We conducted this review to systematically consolidate the available evidence regarding the association between the Ty/HDLc ratio and the prevalence and severity of periodontitis, and to identify knowledge gaps to guide future research regarding the Ty/HDLc ratio and periodontitis.

## Methods

We registered the protocol for this systematic review in the International Prospective Register of Systematic Reviews (PROSPERO) (Registration ID: CRD420251054047) and reported following the PRISMA 2020 guidelines [36].

### Eligibility criteria

We used the PEO model to formulate the research question: Population: adolescents and adults (individuals of age ≥10 years); Exposure: the Ty/HDLc ratio assessed either as a continuous variable or in categorised form; and Outcome: the prevalence or severity of periodontitis assessed through parameters such as community periodontal index (CPI), bleeding on probing (BOP), probing pocket depth (PPD), clinical attachment loss (CAL), or radiographic bone loss. The review question: “Is there an association between the serum triglycerides to high-density lipoprotein cholesterol ratio (Ty/HDLc ratio) and the prevalence or severity of periodontitis in adolescents and adults?”

We included all observational studies in adults or adolescents that reported a quantitative measure of the Ty/HDLc ratio and its association with periodontitis. Adolescents (≥10 years) were included in this review to capture early life stages where long-term lifestyle habits that lead to metabolic alterations may first develop. Similarly, early manifestations of periodontal inflammation and attachment loss often begin in this age group, providing insight into the initial link between metabolic and periodontal health. Including adolescents, therefore, broadens the understanding of this association across the course of life and enhances the generalizability of the findings. Studies in children below the age of 10 years, interventional studies, animal studies, in vitro studies, reviews, editorials, and case reports were excluded.

### Search strategy

Before the formal search strategy, we conducted a preliminary search in ‘Google Scholar’ using the keywords ‘triglycerides/HDL-C ratio’ and ‘periodontitis’ to check the scope of available literature. For the formal search, we chose four databases: PubMed/MEDLINE, Embase, Scopus, and Web of Science. We used keywords related to ‘triglycerides/HDL-C ratio’, ‘dyslipidemia’, ‘periodontitis’, and ‘oral health’ and combined them using Boolean operators to construct the search strings tailored for each database. After all the reviewers approved the search strategy, the search was completed on May 27, 2025, and studies published up to that date were included. Date or language restrictions were not applied. We also searched the citation list of relevant review articles for eligible studies. The details of keywords, search strings, and the date of search for each database are provided as a supplementary file.

### Study selection

After the formal search, we imported all the records into the Rayyan web app [37] for duplicate removal and screening. After removing the duplicates, S.S. and S.K. independently screened the titles and abstracts of the remaining unique records. Any differences between the reviewers were resolved through discussion with the third reviewer (R.A.). Records that passed the title/abstract screening phase were selected for full-text screening. The full-text files for the selected records were retrieved, and S.S. and S.K. independently reviewed the full-text files to verify whether they met our eligibility criteria. One record that was selected for full-text screening was published in Spanish and was translated to English independently by S.S. and S.K. using ‘Google Translate’. Records that did not satisfy the eligibility criteria were excluded, and the specific reason for exclusion was documented. The articles that passed the full-text screening phase were selected for the final synthesis and moved to the data extraction phase.

### Data extraction

S.S. and S.K. independently collected the relevant data from the final set of included articles into an Excel-based data extraction form. Data regarding the following variables were collected: study characteristics (author, year, country, study design, sample size); participant characteristics (study population, mean age, gender distribution); exposure measures (Ty/HDLc ratio continuous/categorical); outcome measures (periodontitis definition, prevalence/severity measured, clinical parameters used); effect measures (odds ratios, risk ratios, correlation coefficients, p-values); confounding factors adjusted for; and key findings of the study. Any differences in the collected data between the two reviewers were resolved through discussion. S.S. and S.K. independently uploaded the Spanish article to ‘Google Translate’ and translated the article into English to extract the relevant data independently. The data extracted from the translated article was cross-verified by a third reviewer, R.A.

### Assessment of study quality

S.S. and S.K. used the Joanna Briggs Institute (JBI) Critical Appraisal Checklist for analytical cross-sectional studies [38] to assess the quality of the included studies independently. The checklist consists of eight question items that assess different domains of the study. Each question item in the checklist is answered as either ‘Yes’, ‘No’, or ‘Unclear’. Each ‘Yes’ response was given 1 point, and ‘No’ and ‘Unclear’ were given 0 points. Based on the responses for all 8 items, the overall study quality was rated as low risk (≥6 points), moderate risk (3–5 points), or high risk of bias (≤2 points). Any differences were resolved through discussion.

### Data synthesis

There was significant heterogeneity among the included studies regarding Ty/HDLc ratio assessments (continuous variables and categorical variables); periodontitis assessments (CPI, Staging of periodontitis, CDC/AAP classification); reported effect measures (odds ratios, correlation coefficients, standardised coefficients); and the presence of partially overlapping study populations. The partial overlap in study populations between the two KNHANES-based studies was identified by comparing the survey years reported in the studies (KNHANES 2012-2014 and KNHANES 2013-2015).

We conducted an exploratory random-effects meta-analysis using the restricted maximum-likelihood method (R 4.5.1; meta package) with studies reporting comparable odds ratios to estimate the pooled odds ratio and its 95% confidence interval (95% CI). Heterogeneity was expressed using the *I*² statistic, *τ*², and Cochran’s Q test. *I*² values of 25%, 50%, and 75% were interpreted as low, moderate, and high heterogeneity, respectively. Both the KNHANES-based studies were retained in the descriptive narrative synthesis but were not pooled to avoid duplication bias.

Observational studies often differ in analytic design, covariate adjustment, outcome definitions, and effect measures, making pooled estimates potentially misleading; methodological reviews have cautioned that inconsistent effect metrics and substantial clinical and methodological heterogeneity can bias meta-analytic results and compromise interpretability [39–41]. Hence, we chose to keep the descriptive narrative synthesis as the primary approach and the meta-analysis as an exploratory tool to discuss the general direction of the association between the Ty/HDLc ratio and periodontitis.

## Results

### Study selection

**Fig. 1:**
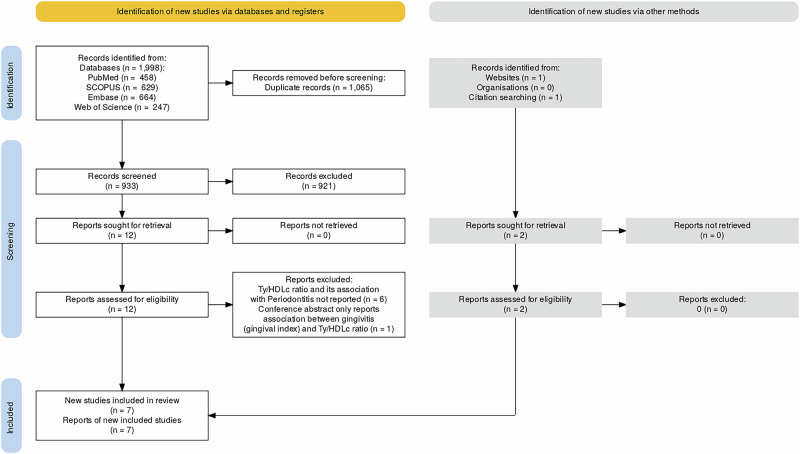
PRISMA flowchart of study selection process. Ty/HDLc Triglycerides/HDL Cholesterol ratio.

The search across four databases (PubMed, SCOPUS, Embase, and Web of Science) yielded a total of 1998 records. After duplicate removal, 933 unique records were screened for title and abstract. Based on titles/abstracts, 921 records were excluded, and 12 records were selected for full-text screening. Additionally, 2 records were identified through other websites (Google Scholar) and citation searching. A total of 14 records were considered for the full-text screening phase. Six articles were excluded as they did not report the Ty/HDLc ratio or its association with periodontitis [42–47]. One study, published as a conference abstract, was excluded as it assessed only gingival inflammation (using the Gingival Index) and did not measure periodontitis as defined in the eligibility criteria [48]. A total of 7 studies [49–55] satisfied our eligibility criteria and were chosen for the final synthesis (Fig. 1).

### Study characteristics

All seven included studies were published between 2018 and 2024 and had a cross-sectional study design, and studied diverse population groups from Brazil [50, 53, 54], Mexico [55], South Korea [49, 51], and Taiwan [52]. Both adolescents and adults were investigated in the included studies, and the mean age of the study population ranged from 17 years [53] to 53 years [50]. Sample sizes ranged from 69 university students [55] to nationally representative surveys involving 13,584 participants [51]. The gender distribution was generally balanced in all studies, except the study by Tsai et al. (2023), which investigated only males [52].

### Exposure and outcome assessment

All included studies measured the Ty/HDLc ratio using fasting serum samples. Three studies assessed the ratio as a continuous variable [52, 53, 55]; three studies as a categorical variable [49–51]; and one study as a composite measure (latent variable) called Insulin Resistance Phenotype [constructed using the Ty/HDLc ratio, very low-density lipoprotein cholesterol (VLDL) concentration, and the Triglyceride-Glucose Index (TyG index)] [54].

Three studies used the Community Periodontal Index (CPI) [49, 51, 55]. One study assessed periodontitis using the CDC/AAP classification to classify periodontitis as mild, moderate, severe or no periodontitis [50]. One study classified periodontitis into stages as proposed by the 2017 World Workshop classification of periodontitis [52]. Two studies used composite measures called latent variables: ‘Initial Periodontitis’ latent variable was constructed using PPD, BOP, and CAL [53] and ‘Chronic Oral Disease Burden’ latent variable was constructed using the number of decayed teeth, the number of teeth with visible plaque, BOP, PPD, and CAL [54].

### Summary of findings

All seven included studies consistently found a positive association between the Ty/HDLc ratio and the prevalence and/or severity of periodontitis (Table 1). Four studies reported odds ratios for the association between the Ty/HDLc ratio and periodontitis [49–52].

**sTable 1:** Summary of findings.

Study	Country	Sample size	Age (mean or range)	Ty/HDLc measurement	Periodontitis definition	Effect estimate (value) with 95% CI	*P*-value	Confounders adjusted for	Key findings
Kwon et al., 2018	Korea	12,249	Men 44.4 ± 0.3 years; Women 46.3 ± 0.3 years	Tertiles Men: T1 (<2.0), T2 (2.0 to 3.6), T3 (≥3.6) Women: T1 (<1.3), T2 (1.3 to 2.4), T3 (≥2.4)	CPI score ≥3 with at least one site positive for periodontitis	Men: T3- (OR: 1.474; 95% CI: 1.220–1.780) Women: T3- (OR: 1.259; 95% CI: 1.041–1.522)	Men (*p* < 0.001) Women (*p* < 0.001)	Age, Waist circumference, Systolic BP, FBS, Smoking, Alcohol, Physical activity, Household income, Frequent tooth brushing, dyslipidemia medication	The Ty/HDLc ratio was independently associated with periodontal disease in Korean adults.
Gomes-Filho et al., 2021	Brazil	1011	53.15 ± 14.63 years	Categorical Group 1: Ty/HDLc ratio < 2.3 Group 2: Ty/HDLc ratio ≥ 2.3	CDC/AAP definition	Periodontitis and Ty/HDLc ≥ 2.3= (OR: 1.47; 95% CI: 1.02–2.14) Severe periodontitis and TG/HDL-C≥2.3 (OR: 1.57; 95% CI: 1.03–2.37)	Prevalence *p* = 0.04 Severity *p* = 0.03 *p* = 0.03 *p* = 0.04	Age, sex, education, smoking, dentist visits, liver disease, FBS, BP, dyslipidemia medication.	The findings of this study showed a positive association between both moderate and severe periodontitis and a Ty/HDLc ratio ≥ 2.3.
Lee et al., 2022	Korea	13,584	50.1 ± 15.8 years	Categorical Quartiles Q1 < 1.32, 1.32 ⩽ Q2 < 2.18, 2.18 ⩽ Q3 < 3.64 3.64 ⩽ Q4	CPI score ≥3 with at least one site positive for periodontitis	Q4- (OR: 1.23; 95% CI: 1.02–1.48) Men: Q4- (OR: 1.44; 95% CI: 1.08–1.92) Women: Q4- (OR: 1.09; 95% CI: 0.84–1.41)	*p* < 0.001 Men: *p* = 0.001 Women: *p* = 0.115	Age, sex, BMI, smoking, alcohol, exercise, Hypertension, Diabetes, Dyslipidemia, and oral hygiene habits.	The study found that the participants in the highest quartiles for the Ty/HDLc ratio had a significantly higher prevalence of periodontitis than the participants in the lowest quartiles.
Ladeira et al., 2022	Brazil	405	17–18 years	Continuous	Initial Periodontitis (latent variable using CAL, PPD, and BOP).	Standardised Coefficient= 0.0130, SE = 0.054	*p* = 0.016	Socioeconomic status, smoking, alcohol, adiposity	Higher Ty/HDLc values were associated with Initial Periodontitis in adolescents
Ladeira et al., 2023	Brazil	2515	18–19 years	Continuous latent variable (Insulin resistance phenotype)	Chronic oral disease burden (latent variable)	Standardised Coefficient= 0.052, SE = 0.025	*p* = 0.033	Socioeconomic Inequalities, Behavioural Risk Factors (smoking, alcohol abuse, and added-sugar consumption), obesity,	The ‘Insulin Resistance Phenotype’ and ‘Chronic Oral Disease Burden’ was positively associated.
Tsai et al., 2023	Taiwan	1111	30.58 ± 5.81	Continuous	2017 World workshop AAP- Periodontitis (Stages 1, 2 and 3)	Stage 3: (OR: 1.10; 95% CI: 1.04–1.16)	*p* = 0.001	Age, smoking, alcohol intake, abdominal obesity, and hypertension.	Serum Ty/HDLc ratio is dose-dependently associated with the risk of localised periodontitis severity (from stage I to stage III).
García et al., 2024	Mexico	69	19.0 ± 1.0 years	Continuous	Periodontal disease (Gingivitis + Periodontitis): CPI score ≥1	Ty/HDLc ratio vs CPI score Spearman’s correlation coefficient (rho)=0.344	*p* = 0.006	Sex, age, BMI, alcohol use, tobacco use, physical activity, socioeconomic status	A significant correlation was found between the Ty/HDLc index and the CPI score.

*Ty/HDLc* triglycerides/high-density lipoprotein cholesterol ratio, *CDC* Centers of Disease Control and Prevention, *AAP* American Academy of Periodontology, *CPI* Community Periodontal Index, *OR* odds ratio, *CI* confidence interval, *BP* blood pressure, *FBS* fasting blood sugar, *BMI* Body-mass index, *BOP* bleeding on probing, *PPD* probing pocket depth, *CAL* Clinical attachment loss, *SE* standard error.

The study by Gomes-Filho et al. (2021) assessed the Ty/HDLc ratio as a categorical variable and investigated its association with periodontitis in Brazilian adults. They found that individuals with periodontitis were more likely to have a Ty/HDLc ratio ≥ 2.3 compared to healthy individuals free from periodontitis [OR (95% CI): 1.47 (1.02–2.14); *p* = 0.04]. This association was found to be stronger in individuals with severe periodontitis [OR: 1.57 (1.03–2.37); *p* = 0.04] [50].

The studies by Kwon et al. (2018) and Lee et al. (2022) both examined the nationally representative Korean population using the Korea National Health and Nutrition Examination Survey (KNHANES) 2012-14 and the KNHANES 2013-15 datasets, respectively. Both studies assessed the Ty/HDLc ratio as a categorical variable (tertiles and quartiles, respectively), and periodontitis was detected using the community periodontal index (CPI). Kwon et al. (2018) found that men who had a Ty/HDLc ratio ≥ 3.6 (3rd tertile) were more likely to have periodontitis compared to men who had a Ty/HDLc ratio < 2.0 (1st tertile) [OR: 1.47 (1.22–1.78); *p* < 0.001]. Women with a Ty/HDLc ratio ≥ 2.4 (3rd tertile) were more likely to have periodontitis compared to those with a Ty/HDLc ratio < 1.3 (1st tertile) [OR: 1.25 (1.04–1.52); *p* < 0.001] [49]. Lee et al. (2022) found that the individuals in the highest quartile of the Ty/HDLc ratio had significantly higher odds of periodontitis than individuals in the lowest quartile [OR: 1.23 (1.02–1.48), *p* < 0.001]; men having a Ty/HDLc ratio ≥ 3.64 (4th quartile) were more likely to have periodontitis than men who had a Ty/HDLc ratio < 1.32 (1st quartile) [OR: 1.44 (1.08–1.92); *p* = 0.001]. However, women having a Ty/HDLc ratio ≥ 3.64 did not show a significant difference compared to women having a Ty/HDLc ratio < 1.32 [OR: 1.09; (0.84–1.41); *p* = 0.115] [51].

The study by Tsai et al. (2023) assessed the ratio as a continuous variable and investigated its association with periodontitis stages (2017 World Workshop classification of Periodontitis) in a cohort of young male adults of Taiwan; higher Ty/HDLc ratios were associated with more severe localised periodontitis [OR: 1.10 (1.04–1.16); *p* < 0.001]. However, it is important to note that the study found this association only in individuals who are obese and in individuals who have a fasting plasma glucose level of <100 mg/dl [52].

One study reported Spearman’s correlation coefficient for the relationship between the Ty/HDLc ratio and periodontal disease. The study by Garcia et al. (2024) assessed the ratio as a continuous variable and investigated its association with periodontal disease (gingivitis and periodontitis), as measured by the CPI score in Mexican university students aged 18 to 25 years; the study found a moderately positive correlation between the Ty/HDLc ratio and CPI score (Spearman’s *ρ* = 0.344; *p* = 0.006) [55].

Two studies reported Standardised coefficients. The study by Ladeira et al. (2022) reported that the Ty/HDLc ratio was positively associated with the composite measure called Initial Periodontitis (modelled using the number of sites with BOP, PPD ≥ 4 mm, and CAL ≥ 4 mm) in adolescents (17–18-year-olds) (Standardised coefficient 0.13; *p* < 0.016). The study also reported that in subgroup analysis by sex, the association was present only in females (*p* < 0.001) [53]. Ladeira et al. (2023) demonstrated a similar positive association between the composite measures called Insulin Resistance Phenotype (consisting of Ty/HDLc ratio, VLDL concentration, and TyG index) and Chronic Oral Disease Burden (including periodontitis indicators, along with the number of teeth with decay and visible plaque) (Standardised Coefficient 0.053; *p* < 0.033) [54]. Both these studies used Structural Equation Modelling (SEM) to construct composite measures known as latent variables to minimise bias due to errors in measurements [53, 54].

In summary, the findings from these seven studies consistently indicate a positive association between the serum Ty/HDLc ratio and the prevalence and severity of periodontitis in adolescents and adults. Across the included studies, odds ratios for the association between higher Ty/HDLc ratios and periodontitis ranged from ~1.1 to 1.57 (10–57% higher odds of periodontitis). This suggests a modest but consistent increase in the likelihood of periodontitis with higher Ty/HDLc ratios. Although these effect sizes are small to moderate, they are clinically relevant at the population level, particularly given that the Ty/HDLc ratio is routinely measured and reflects broader metabolic health.

An exploratory random-effects meta-analysis including three independent studies: Lee et al. (2022) [51]; Gomes-Filho et al. (2021) [50]; and Tsai et al. (2023) [52] yielded a pooled odds ratio of 1.17 (95% CI: 1.03–1.32; *p* = 0.015) with moderate heterogeneity (*I*² = 41.9%; *τ*² = 0.0056; *Q* = 3.44, *p* = 0.18). Among the four studies that reported odds ratios, two studies (Kwon et al. [49] and Lee et al. [51]) were based on overlapping KNHANES data. Hence, only the more recent study—Lee et al. [51]–with a larger sample size was retained for the exploratory meta-analysis. Given the small number of studies and methodological variability, this quantitative synthesis was used only to estimate heterogeneity; narrative synthesis was retained as primary for the main interpretation.

### Risk of bias

**Fig. 2:**
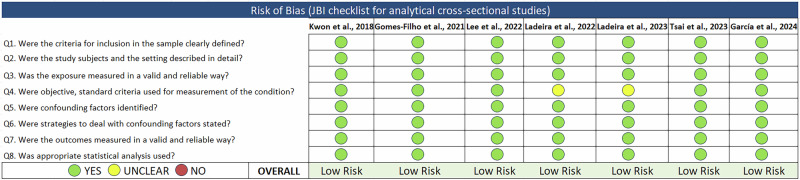
Study quality assessment for the included studies using the Joanna Briggs Institute (JBI) checklist.

All studies adjusted for potential confounders, including age, sex, smoking status, BMI, socioeconomic status, lifestyle factors, comorbidities, and lipid-lowering medications. Notably, two studies used structural equation modelling to account for complex pathways involving adiposity and behavioural risk factors in adolescents [53, 54]. The study quality and risk of bias were assessed using the JBI Checklist for Analytical Cross-Sectional Studies. All seven studies were rated as having a low risk of bias (Fig. 2).

## Discussion

The results across seven cross-sectional studies indicate that a higher Ty/HDLc ratio is positively linked to periodontitis prevalence and severity in adolescents and adults. Despite differences in demographics, periodontitis assessment methods, and geographical settings, the positive association remained consistent. This consistency suggests that the Ty/HDLc ratio may be a clinically relevant, indirect risk marker for periodontitis. The exploratory meta-analysis using three studies: Lee et al. [51]; Gomes-Filho et al. [50]; and Tsai et al. [52] demonstrated a positive association between the Ty/HDLc ratio and periodontitis [OR: 1.17 (1.03–1.32)], with moderate heterogeneity (*I*² = 41.9%). However, a small number of eligible studies, along with methodological and clinical heterogeneity, justify our decision to retain a descriptive narrative synthesis as the primary approach for this systematic review of observational studies [39–41].

Potential confounders such as oral hygiene, smoking, diabetes, obesity, medication use, and socioeconomic status were addressed in most of the included studies. These consistent multivariable adjustments reduce, but do not eliminate, the risk of residual confounding, particularly from unmeasured behavioural and dietary variables. Nevertheless, the persistence of a significant association after adjustment strengthens the likelihood that the Ty/HDLc ratio reflects an independent metabolic correlate of periodontitis rather than a confounded association. Since all included studies were cross-sectional in design, causality or temporal sequence between the Ty/HDLc ratio and periodontitis cannot be understood. It is unclear whether an elevated Ty/HDLc ratio drives periodontal disease or vice versa. We discuss both scenarios for a potential bidirectional link between the Ty/HDLc ratio and periodontitis.

One possible explanation for the link between the Ty/HDLc ratio and periodontitis is a poor lifestyle. Core human lifestyle factors (diet, sleep, physical activity, and stress) play a fundamental role in human health and disease [56–60]. A poor lifestyle—characterised by sub-optimal diet quality and quantity, inadequate sleep, low physical activity, and chronic stress can lead to metabolic dysregulation, dyslipidaemia, obesity, and metabolic syndrome, all of which are reflected in an elevated Ty/HDLc ratio [17, 61, 62]. Periodontitis shows a strong relationship with obesity and metabolic syndrome [9, 10]. Emerging evidence suggests that sub-optimal lifestyle factors also increase the risk of periodontitis through dysbiosis and systemic inflammation [63–69]. Therefore, a poor lifestyle may be the primary factor that initiates a chain of downstream events, starting with metabolic dysregulation and dysbiosis, progressing to chronic systemic inflammation, and culminating in metabolic syndrome, which subsequently increases the risk of periodontitis, T2DM, and CVD. This is evident in the associations between an elevated Ty/HDLc ratio and metabolic syndrome, T2DM, CVD, and periodontitis [19]. And once periodontitis is initiated, it adds to the systemic inflammatory burden and can worsen dyslipidemia through chronic low-grade systemic inflammation, mediated by circulating pro-inflammatory cytokines [70]. A recent meta-analysis confirmed this bidirectional relationship between periodontitis and dyslipidemia [16].

There is a lack of a definitive, clinically applicable threshold value for the Ty/HDLc ratio for periodontal risk assessment in the available evidence. One study by Gomes-Filho et al. (2021) found that individuals with periodontitis were more likely to have a Ty/HDLc ratio of ≥ 2.3 [50]. Also, some of the included studies reported gender differences in the association between the Ty/HDLc ratio and periodontitis. Kwon et al. (2018) reported gender-specific assessments [49]—Women who belonged to the highest tertile had lower Ty/HDLc ratios than men of the highest tertile. In men, the highest prevalence of periodontitis occurred at a Ty/HDLc ratio above 3.6, and in women, it was above 2.4 [49]. Lee et al. (2022) found no significant difference in periodontitis prevalence between women in the highest quartile and women in the lowest quartile [51]. Ladeira et al. (2022), in sub-group analysis by gender, found that the Ty/HDLc ratio was associated with periodontitis only in women [53].

These differences between genders highlight the need for a definitive, clinically relevant, gender-specific threshold for periodontal risk assessment or a universal threshold that accounts for the gender differences. The observed sex differences may arise from both biological and behavioural factors—Estrogen enhances HDL synthesis and exerts anti-inflammatory effects, while testosterone in men is inversely associated with the Ty/HDLc ratio [71, 72]. Behavioural factors such as less frequent dental visits, poorer oral hygiene practices, and higher rates of smoking and alcohol consumption among men may further amplify risk. These differences could partly explain the stronger associations observed among men in certain studies. Future research should perform sex-stratified analyses and account for hormonal and behavioural variables to understand these effects.

This systematic review has several notable strengths: this review is the first to systematically explore the evidence on the association between the Ty/HDLc ratio—a simple, economical, routinely measured and easily calculable lipid parameter—and periodontitis prevalence or severity in adolescents and adults; a comprehensive search across four major databases and citation searching maximised the study capture; the included studies cover diverse population groups from different countries, age groups (including adolescents), enhancing the generalizability of the findings; and, all studies were rated as having a low risk of bias, supporting confidence in the consistency of the observed association.

This systematic review has several limitations. First, the included studies were cross-sectional in design, and the temporal relationship between the Ty/HDLc ratio and periodontitis cannot be inferred. It remains unclear whether derangement in the Ty/HDLc ratio precedes periodontal tissue breakdown or whether periodontal inflammation drives changes in the ratio; however, current evidence supports a bidirectional relationship. Second, there was substantial heterogeneity in how the Ty/HDLc ratio was categorised (continuous variable, tertiles, quartiles, clinical cut-offs, or as a latent variable) and in how periodontitis was defined and measured (e.g. CPI, Staging of periodontitis, CDC/AAP classification, composite variables). These methodological differences in Ty/HDLc quantification and disease classification reduce cross-study comparability and may partly explain the heterogeneity in observed associations. Also, the use of composite variables in two studies—Ladeira et al. (2022) [53] and Ladeira et al. [54]—limits clinical comparability.

Third, the use of automated language translation for one included study may have introduced minor interpretation errors, as we did not formally check for the accuracy of the ‘Google Translate’ tool; however, the main qualitative and quantitative study findings and extracted data were clear and cross-checked independently by three reviewers. Lastly, two studies—Kwon et al. [49] and Lee et al. [51]—used partially overlapping KNHANES datasets, potentially introducing participant duplication and synthesis bias if pooled. However, to minimise this risk, both studies were described narratively, and only the more recent study (Lee et al., 2022) was included in the exploratory meta-analysis. These factors should be considered when interpreting the overall strength and generalizability of the findings.

## Conclusion

Within the limitations of available evidence, the findings of this systematic review suggest that a higher Ty/HDLc ratio is associated with an increased prevalence and severity of periodontitis in both adolescents and adults and highlights the complex interplay between metabolic and periodontal health. The consistency of findings across diverse age groups and population groups indicates the Ty/HDLc ratio’s potential to be a valuable biomarker that reflects an individual’s underlying metabolic health as well as the risk of developing periodontitis.

### Clinical relevance

The serum Ty/HDLc ratio could be a promising biomarker to serve as a simple, practical, cost-effective, and easily calculable indirect risk indicator for periodontitis, especially when integrated into general health screenings, enabling better primary prevention. Dental professionals, in collaboration with medical practitioners, should monitor patients’ lipid profiles to integrate metabolic health into periodontal risk assessment. This can enhance early detection, tailored preventive and therapeutic strategies, which can ultimately improve both oral and systemic health. However, its clinical use should currently be considered adjunctive as evidence remains primarily cross-sectional and cut-off thresholds are not yet standardised.

### Future directions

Given the potential of the Ty/HDLc ratio, prospective cohort and intervention studies should focus on establishing causality and temporal relationship, and a clear and gender-specific cut-off for periodontal risk assessment. Future research should also adopt standardised Ty/HDLc assessments and periodontitis case definitions along with comprehensive adjustment for confounders. Interventional studies should explore the potential of periodontal therapy in improving an individual’s metabolic health and whether targeting an individual’s metabolic health translates into improvements in periodontal health outcomes. Future research should also explore the underlying mechanisms that link metabolic health and periodontitis to deepen our understanding of the link between oral and systemic health.

## Supplementary information

Search Strategy

PRISMA checklist
