# Supplementary material for: Triglycerides to high-density lipoprotein cholesterol ratio and its association with periodontitis—a systematic review
Source: BDJ Open. 2025 Dec 4;11:90. doi: 10.1038/s41405-025-00381-1 (PMC12678417; doi:10.1038/s41405-025-00381-1)
Supplement: Supplementary file 1 — Search Strategy [file 41405_2025_381_MOESM1_ESM.pdf]

Triglycerides to High-Density Lipoprotein Cholesterol Ratio and Its Association with Periodontitis- A Systematic Review

Search terms

“Triglycerides to High density lipoprotein cholesterol ratio”	Periodontitis
“Triglycerides-to-High density lipoprotein cholesterol ratio”	“Periodontal inflammation”
“TG-to-HDL-C ratio”	“Gingival inflammation”
“triglyceride/high density lipoprotein cholesterol ratio”	Gingivitis
“TG/HDL-C ratio”	“Periodontal disease”
“Triglyceride to high density lipoprotein cholesterol ratio”	“Oral disease”
“Triglyceride-to-high density lipoprotein cholesterol ratio”	“Oral health”
Dyslipidemia	“Oral cavity”
Hypertriglyceridemia	“Chronic periodontitis”
	“Gingival disease”
	“Periodontal health”
	“Gingival health”
	“Dental health”
	“Generalised periodontitis”
	“Localized periodontitis”

Search string

“Triglycerides to High density lipoprotein cholesterol ratio” OR “Triglycerides-to-High density lipoprotein cholesterol ratio” OR “TG-to-HDL-C ratio” OR “triglyceride/high density lipoprotein cholesterol ratio” OR “TG/HDL-C ratio” OR “Triglyceride to high density lipoprotein cholesterol ratio” OR “Triglyceride-to-high density lipoprotein cholesterol ratio” OR Dyslipidemia OR Hypertriglyceridemia AND Periodontitis OR “Periodontal inflammation” OR “Gingival inflammation” OR Gingivitis OR “Periodontal disease” OR “Oral disease” OR “Oral health” OR “Chronic periodontitis” OR “Gingival disease” OR “Periodontal health” OR “Gingival health” OR “Dental health” OR “Generalised periodontitis” OR “Localized periodontitis”

1. PUBMED, MEDLINE

Search String:

("Triglycerides to High density lipoprotein cholesterol ratio" OR "Triglycerides-to-High density lipoprotein cholesterol ratio" OR "TG-to-HDL-C ratio" OR "triglyceride/high density lipoprotein cholesterol ratio" OR "TG/HDL-C ratio" OR "Triglyceride to high density lipoprotein cholesterol ratio" OR "Triglyceride-to-high density lipoprotein cholesterol ratio" OR Dyslipidemia OR Hypertriglyceridemia) AND (Periodontitis OR "Periodontal inflammation" OR "Gingival inflammation" OR Gingivitis OR "Periodontal disease" OR "Oral disease" OR "Oral health" OR "Chronic periodontitis" OR "Gingival disease" OR "Periodontal health" OR "Gingival health" OR "Dental health" OR "Generalised periodontitis" OR "Localized periodontitis")

PUBMED/MEDLINE: 458 results; Searched on: 27th May, 2025 (9:15); .nbib file generated

2. SCOPUS

(TITLE-ABS-KEY ("Triglycerides to High density lipoprotein cholesterol ratio" OR "Triglycerides-to-High density lipoprotein cholesterol ratio" OR "TG-to-HDL-C ratio" OR "triglyceride/high density lipoprotein cholesterol ratio" OR "TG/HDL-C ratio" OR "Triglyceride to high density lipoprotein cholesterol ratio" OR "Triglyceride-to-high density lipoprotein cholesterol ratio" OR dyslipidemia OR hypertriglyceridemia) AND TITLE-ABS-KEY (periodontitis OR "Periodontal inflammation" OR "Gingival inflammation" OR gingivitis OR "Periodontal disease" OR "Oral disease" OR "Oral health" OR "Chronic periodontitis" OR "Gingival disease" OR "Periodontal health" OR "Gingival health" OR "Dental health" OR "Generalised periodontitis" OR "Localized periodontitis"))

SCOPUS: 629 results; Searched on: 27th May, 2025 (9:21); RIS file generated

3. EMBASE

('triglycerides to high density lipoprotein cholesterol ratio' OR 'triglycerides-to-high density lipoprotein cholesterol ratio' OR 'tg-to-hdl-c ratio' OR 'triglyceride/high density lipoprotein cholesterol ratio' OR 'tg/hdl-c ratio' OR 'triglyceride to high density lipoprotein cholesterol ratio' OR 'triglyceride-to-high density lipoprotein cholesterol ratio' OR dyslipidemia OR hypertriglyceridemia) AND (periodontitis OR 'periodontal inflammation' OR 'gingival inflammation' OR gingivitis OR 'periodontal disease' OR 'oral disease' OR 'oral health' OR 'chronic periodontitis' OR 'gingival disease' OR 'periodontal health' OR 'gingival health' OR 'dental health' OR 'generalised periodontitis' OR 'localized periodontitis')

EMBASE: 664 results; Searched on: 27th May, 2025 (9:43); RIS file generated

4. WEB OF SCIENCE

https://www.webofscience.com/wos/woscc/summary/2260410c-e4eb-4125-940d-99d0d7978b85-01649d1fc2/relevance/1

(ALL=("Triglycerides to High density lipoprotein cholesterol ratio" OR "Triglycerides-to-High density lipoprotein cholesterol ratio" OR "TG-to-HDL-C ratio" OR "triglyceride/high density lipoprotein cholesterol ratio" OR "TG/HDL-C ratio" OR "Triglyceride to high density lipoprotein cholesterol ratio" OR "Triglyceride-to-high density lipoprotein cholesterol ratio" OR Dyslipidemia OR Hypertriglyceridemia)) AND ALL=(Periodontitis OR "Periodontal inflammation" OR "Gingival inflammation" OR Gingivitis OR "Periodontal disease" OR "Oral disease" OR "Oral health" OR "Chronic periodontitis" OR "Gingival disease" OR "Periodontal health" OR "Gingival health" OR "Dental health" OR "Generalised periodontitis" OR "Localized periodontitis")

WOS: 247 results; Searched on: 27th May, 2025 (9:48); RIS file generated
